# Remarks on Intra-Uterine Polypi, Etc.; with Three Cases

**Published:** 1876-02

**Authors:** A. Reeves Jackson

**Affiliations:** Surgeon-in-Chief of the Woman’s Hospital of the State of Illinois; Lecturer on Diseases of Women in Rush Medical College; Chicago


### SOME REMARKS ON INTRA-UTERINE POLYPI, WITH SPECIAL REFERENCE TO THEIR DIAGNOSIS AND SURGICAL TREATMENT; WITH THREE CASES.

BEING THE REPORT OF THE GYNECOLOGICAL SUB-SECTION OF THE SECTION ON OBSTETRICS AND DISEASES OF WOMEN AND CHILDREN.

(Read before the Chicago Society of Physicians and Surgeons, Nov. 22, 1875.)

BY A. REEVES JACKSON, M.D.,

Surgeon-in-Chief of the Woman's Hospital of the State of Illinois; Lecturer on Diseases of Women in Rush Medical College.

MR. PRESIDENT AND GENTLEMEN:

It would be impossible, without taxing your time and patience to an unwarrantable degree, to make a report which would adequately cover the ground now occupied by Gynecology, a department which has attained a position scarcely second in extent and importance to the other great practical branches of medicine. I have therefore selected from this wide domain a single subject, which, while free of intrinsic interest, will serve to exemplify some of the advances which have been made in recent years in this field of surgery.

A polypus of the womb is an outgrowth of some portion of the uterine tissue, which remains attached to the wall of the organ either by a broad base or a defined peduncle. It is therefore a local hypertrophy of some of the normal structures composing the organ. These growths vary greatly in size, being sometimes not larger than a grape seed, and at others larger than a cocoa-nut. They grow from any portion of the uterus ; from its external surface or from its interior. In the remarks which follow, I will confine myself to the latter class, namely, those which have their origin in some portion of the cavity of the organ, or project into it, and which are still retained within its limits. This will exclude from our consideration not only other morbid growths to which the term polypus is sometimes applied, such as retained portions of placenta or of fœtal remains, fibrinous polypi or blood-clot, cauliflower excrescence and carcinomatous growths, although these may and frequently do assume the polypoid form and give rise to symptoms identical with those of true polypus, but also true polypi which project from the peritoneal surface of the womb and which arise from the external os and cervical portion.

Intra-uterine polypi may take their origin from any part of the interior of the organ—the fundus, the body, or the cervix. As they increase in size they usually pass downwards through the os uteri into the vagina, and they may even protrude beyond the vulva. When they have emerged from the os uteri and are discoverable in the vagina, their diagnosis and treatment become comparatively easy and simple.

*Varieties.*—Uterine polypi are of three kinds—the mucous, the cystic, and the fibrous.

The mucous polypi consist of the cellular or connective tissue of the mucous membrane. They are commonly pedunculated, the peduncle being formed by an elongation of the membranous tissue drawn downward by the growing polypus. They are usually of small size, being rarely larger than a pea, although I have seen one as large as an English walnut, and Dr. Atthill gives an account of one which he saw in a patient twenty-four hours after delivery, which had attained the size of an orange. It was attached by a long slender pedicle just inside the os uteri and hung partially outside the vagina. These polypi are highly vascular, of a bright pink, or pinkish gray color, and commonly of a soft gelatinous consistence. They may grow from any part of the cavity of the womb, but are most frequently found attached to the cervix.

The cystic or glandular polypi also have their origin in the mucous membrane, or rather in the glandulur structures imbedded in it. Their attachment is always sessile, and they rarely exceed a filbert in size. Generally they are much smaller, and when they exist in great numbers, as they sometimes do, the aggregation resemble a vegetating mass surrounding the os uteri like a fringe. They are formed from the enlarged utricular or Nabothian glands, although they are thought to be sometimes new formations. They are whitish or pearl-colored, and consist of clear, tenacious mucus resembling the white of an uncooked egg, enclosed in these delicate walls which are easily ruptured. Their usual place of origin is the cervical canal.

The fibrous polypi grow from the muscular or submucous tissue of the uterus. They consist of the same anatomical elements, in varying proportions, as compose the parenchyma of the womb, and sometimes attain a very large size. A fibrous polypus may be regarded as simply a fibrous tumor in an advanced stage of development. The two are identical in structure ; either may be surrounded by a capsule of condensed cellular tissue, and the symptoms produced by them are the same. A

fibroid tumor of the sub-mucous variety, partly by increasing in size more than the parts around it, and partly, also, by the contraction of the surrounding muscular fibres, projects in the direction of the least resistance—that is, into the uterine cavity. These forces continuing to act, the tumor finally becomes pyriform and pedunculated from its own weight, and its largest portion hangs in the distended cavity of the uterus. In other cases its attachment remains sessile. In either case it soon occludes the canal to some extent, either at the internal os or at some part of the cervical canal, and, operating in the manner of a ball-valve, prevents the free exit of any fluid that may be poured into the cavity above it. The consequent distension of the uterine walls causes contractions, sometimes of a very powerful character, and the polypus, when pedunculated, is forced downwards and may eventually pass into the vagina. This process of distension is from the first facilitated by the loose nature of the attachment of these muscular tumors to the surrounding uterine tissue.

Fibrous polypi generally arise from the fundus or body of the uterus, although they are sometimes attached to the cervix. In the majority of cases they are solitary, but Klob states that two are sometimes found together, their sides being flattened by contact with each other; and Dr. George H. Kidd* has given the details of a very remarkable case, in which he removed twenty-nine fibrous polypi from the interior of the cavity of the body of the uterus of an unmarried woman, aged fifty-six years, at four operations—all within a period of ten months. This case is certainly quite unique.

The pressure of a fibrous polypus in the cavity of the womb stimulates the organ to increased growth, and its walls become thickened and elongated, so that the fundus may frequently be felt above the pubes, and even as high as the umbilicus. It may incline to either side, and may

* *Dublin Quarterly Journal of Medical Science*, Feb., 1869.

be regular or irregular in outline, according to the shape of the growth within.

*Symptoms.*—The symptoms which denote the pressure of polypus are chiefly three—hæmorrhage, leucorrhœa, and pain. Usually the first symptom which attracts the notice of the patient is a profuseness of discharge at the catamenial periods. At first she may scarcely observe the change, but after a time the periods increase in length and the intermenstrual times become shortened; and, by and by, she has a sanguineous discharge on making any extra exertion, so that in some instances there is an almost constant hæmorrhagic flow.

Alternating with this discharge is the next most common symptom, leucorrhœa. This is generally abundant, and frequently offensive. Sometimes it is clear, transparent, ropy and tenacious, and may be seen hanging from the os uteri like a mass of jelly; at others, it is yellowish, brownish or greenish, and occasionally streaked with blood. Sometimes the discharge is of a thin, watery character, tinged with blood. This is especially likely to accompany the pressure of the small cystic polypi that grow in the canal of the cervix, and produce a patulous condition of the os.

Under such a continual drain, the health of the patient soon fails; she becomes feeble, pale and cachectic; digestion is deranged; she has pains in the back, and a sense of weight and dragging in the pelvis.

Pain, although a frequent accompaniment of polypus, is not so generally present as the symptoms already mentioned. When the polypus is so situated as to obstruct the exit of fluid from the cavity of the uterus, very severe pain of an expulsive character is experiencd during the menstrual flow; and similar pains may occur at other times from the retention of mucous and serous fluids. Pain is also felt in many cases behind the pubis, and in the neighborhood of one or other ovary—never, in my experience, in *both.* The pain varies in degree from the slightest soreness to the most severe uterine colic.

The gravity of the symptoms produced by uterine polypi is by no means proportionate to the size of the growth; for, while in some cases of very large polypi the hæmorrhage, leucorrhœa and pain may all be slight, in others, a polypus no larger than a currant may cause the most exhausting leucorrhœa or even fatal hæmorrhage.

When, in connection with spasmodic or intermittent pains resembling those attendant upon labor or abortion (these conditions being excluded), there are frequent or copious hæmorrhages and leucorrhœa, with or without enlargement of the womb, we may suspect the presence of polypus, but with these rational symptoms only we can do no more than suspect it. Nothing but a physical exploration can enable us to decide the point definitely; and when the symptoms enumerated can not be otherwise accounted for, and are not controllable by the use of ordinary means, an examination of the pelvic organs should be promptly made.

*Diagnosis.*—As already stated, uterine polypi, when they have descended beyond the os uteri, are easily discovered both by touch and sight, and they are as easily treated; but it is a very different matter when they are still within the uterine cavity. Occasionally, under favorable circumstances—for example, during an attack of bleeding when the cervix is greatly relaxed—the polypus may descend so low as to be on the point of emerging from the os uteri, and may then be perceived by the finger, or even by the use of the bivalve speculum, which expands the os when its blades are separated. But in most cases the diagnosis can only be made with certainty by artificially dilating the cervical canal sufficiently to permit the introduction of the finger.

The means now generally used for dilating the cervix are expansible tents made of prepared sponge, or of the laminaria digitata, or sea tangle. In this country the sponge is most commonly used, while in Europe preference appears to be given to the laminaria. For myself if restricted to the use of one only of these agents, I would prefer the sponge tent, because of its superior dilating power when well made; but in most cases I employ both.

It has been urged, as the chief objection to the use of the sponge tent, that it becomes horribly offensive after lying in the genital tract a few hours; but, inasmuch as this unpleasant odor can be prevented in great degree, by incorporating with the tent either permanganate of potash or a solution of carbolic acid, this drawback should not stand in the way of its employment. My objection to the sponge tent is, I think, a much more serious one; its use almost invariably results in the removal of the epithelium from the mucous membrane, leaving a large denuded surface, which may become an avenue for the admission of septic material into the system.

In order to avoid this, I adopt the following plan: instead of introducing as large a sponge tent as the cervical cavity will admit, as is the common custom, I prefer to use a smaller one, and then insert by its side, one after another, small slips of sea-tangle, sufficient in number to surround, if possible, the sponge. In this manner a much larger quantity of dilating material can be used than is possible with a single sponge tent, or with a number of laminaria tents used without the sponge, and, at the same time, the mucous membrane is protected from the injurious contact of the sponge. To accomplish this proceeding properly, the sponge tent should be slightly coated with a mixture of melted wax and lard—one part to three—in order that it may not absorb moisture and commence to expand too quickly.

It is quite important that sponge tents should be well made, and of properly selected material. Many of those in the market, although very elegant in appearance, are almost worthless as dilating agents, and I have sometimes been greatly disappointed at finding them, twenty-four hours after their introduction, scarcely larger than when first inserted. In order to be efficient they should not be made of the finest and closest sponge, nor, on the other hand, of the very coarse or honeycomb variety ; the first lacks resiliency, and the other lacks power. Good, sound, unbleached sponge, of medium texture, is the best for the purpose. Sponge tents should not be saturated with mucilage of gum Arabic, as is still directed by some authorities; but, instead of this, they should, before being compressed, be soaked in a weak solution of carbolic acid.

When the first attempt fails, as it sometimes does, to effect a sufficient degree of dilatation, rather than repeat the introduction of the tents, I prefer to use the rubber dilator of Molesworth. From frequent use of this instrument, I can bear witness to its safety and efficiency. By its aid, after the cervix has been softened and relaxed by the tents, we can usually, in a few minutes, produce all the additional dilatation necessary.

The cervix having been sufficiently opened, the index finger may be passed into it, and if firm downward pressure be made at the same time upon the fundus through the hypogastrium, we can, in many cases, explore the uterine cavity beyond the os internum, and be enabled to detect the presence of an existing polypus, even of the smallest size.

If the polypus be of the larger kind, the finger will probably pass around it, between it and the cervix, on every side, showing that its attachment is higher up, the distance being determinable in some measure by the degree of mobility of the growth.

In the case of very obese patients, or where, from any other cause, the finger cannot be made to penetrate sufficiently far, we may usually succeed by inducing anæsthesia, and introducing the hand into the vagina.

In examining for polypi, it is well to bear in mind that they are sometimes intermittent in their appearance. A case came under my observation, several years ago, in which a mucous polypus, about the size of a cherry, could only be seen during the menstrual periods. At those times it protruded slightly beyond the os uteri, but at other times it receded within the cervical canal, and could neither be seen nor felt. Several writers have reported similar instances. Hence, it is better, in a suspected case, to make the examination during the menstrual flow, or an attack of metrorrhagia. The polypus is not only more likely to descend lower at such times, but the relaxation of the os and cervix are also much greater, and are more likely to admit the tip of the finger.

The uterine sound is commonly not of much use in enabling us to differentiate a polypus from other growths or conditions. It may teach us definitely that the cavity of the uterus is lengthened, and inform us as to its direction, but it is only the fibrous polypus that causes notable enlargement of the womb, and the same result is produced by an imbedded fibrous tumor.

A few years ago, Dr. Kidd called attention to the fact, that in several cases observed by him, the polypus caused a bulging of the uterine wall opposite the point of attachment; and he maintained that if it should be found, after more extended observation, to be a law, that an intra-uterine pedunculated polypus in its development produced a bulging of the opposite side, while an interstitial or submucous fibrous tumor caused, as is well known, a bulging of the wall in which it is developed, the fact would be of great practical importance. For a knowledge of it would enable us, by the use of the sound alone in many cases, to diagnosticate between these two forms of neoplasm—that is to say, where the sound passed on the side of the bulging, it would show that the growth was a polypus, and where it passed on the other side, it would indicate the presence of a non-pedunculated growth. But such is not the law; or, if it be, there are so many exceptions to it as to make it valueless for the purpose indicated. I have myself seen two cases of polypus in which the bulging corresponded with the point of attachment.

There is occasionally great difficulty in introducing the sound, and sometimes this cannot be done at all. This happens when the polypus drags the fundus very strongly forward or backward, in one case throwing the os uteri upward into the hollow of the sacrum, and in the other behind the symphisis pubis.

I have never been able to verify the claim made by some gynecologists, that by the use of the sound we can ascertain the fact of mobility of an intra-uterine polypus; and I have the belief that such a notion has arisen from an erroneous sense of touch. When a polypus is sufficiently large to be perceived at all by an insensitive metallic instrument, it is in such close contact with the walls of the womb as not to be readily moved about; and, notwithstanding the fact that they are frequently so represented in the books, uterine polypi do not hang in the cavity of the womb, as does the clapper in a bell, capable of dangling from one side to the other.

*Treatment.*—Uterine polypi sometimes disappear spontaneously. This may occur in two ways. First, the weight of the polypus by its traction upon the pedicle may cause the latter to become thinner and thinner until the growth drops off; secondly, ulceration of a destructive character commences in that portion of the mucous membrane covering the pedicle, and the latter becomes softened and gangrenous. When this latter result happens, the symptoms strongly resemble those of cancer.

But it is not our duty to wait for any such spontaneous cure, which very rarely, and usually never occurs. When we have ascertained the presence of a polypus it is our duty to remove it, if removal be practicable. The operation may be a very simple affair, or it may be one of the most difficult in surgery. If the growth be small and low down in the cervix, or, if the pedicle be attenuated and readily reached, it will be sufficient to seize it with a pair of suitable forceps and twist it round and round until it drops off; or, in such cases, the pedicle may be snipped with scissors. When forceps are used, they should be strong both in blade and joint.

But these modes of procedure only succeed when the pedicle is small. When it is large and thick, other means must be used. Of these, the best and the one most usually available is the écraseur, armed with a single soft iron wire. Dr. Kidd and Dr. Atthill prefer a steel wire—a piece of piano-forte string. They consider the steel quite as manageable as the iron after its introduction to the uterine cavity, and it is certainly much stronger. In a single case in which I tried it, I did not find it so convenient as the soft wire, and, indeed, was obliged to change it for the latter. The wire should be carefully selected, and should be free from rust-spots and any other defect. It should be adequate in size and strength for the work it has to do. It is not only exceedingly annoying to have a wire break inside the uterine cavity, after it has been placed about the peduncle of a polypus with, perhaps, great difficulty, but such a mishap is also dangerous, for it is almost impossible to withdraw it without seriously wounding the interior of the womb. Likewise, the instrument should be strong, and the screw furnished with sufficient leverage to tighten the wire easily. The more power we have at command, the more gently and steadily it can be employed.

The operation for removal of large polypi should always be done under anæsthesia. The fatal result in one of the cases I will relate further on, occurred, I believe, indirectly from neglect of this condition. The pain and tension upon the nervous system proved too great for the patient's power of endurance, and prevented the proper completion of the operation.

The patient, then, having been brought fully under the effect of ether or chloroform, the operator should seize the anterior lip of the uterus firmly, with a strong vulsellum, and by steady traction aided by supra-pubic pressure, the cervix should be brought down to the vulva. Then, giving the vulsellum in charge of an assistant he (the operator) should pass the index finger into the cervix and ascertain, accurately, the size and place of attachment of the peduncle. A second strong vulsellum is next to be passed into the cervical canal and affixed as high as possible on the polypus. This being done, the wire loop of the écraseur, passing over the handle of the instrument and guided by the finger, is placed around the peduncle at the proper point and slowly tightened by turning the screw. This process should be continued, steadily and gradually, until the peduncle is cut through, when the écraseur, vulsellum and polypus may be brought away together.

Before releasing the hold of the vulsellum upon the cervix, the finger should again be passed in and the uterine cavity thoroughly explored, to ascertain whether more polypi be present. If so, they should be at once removed; if not, the whole interior of the womb should be brushed over with a solution of perchloride of iron in glycerine, and the patient placed in bed.

In applying the loop of wire around the pedicle it is neither necessary nor desirable that it should enclose the latter very near its attachment to the uterine wall—not nearer than a half inch or an inch. It is undesirable because the proceeding is then more likely to be followed by pain and inflammation; and it is unnecessary because it has been repeatedly demonstrated that the removal of any considerable portion of a polypus is as surely curative as though the whole be taken away.

Occasionally much difficulty is experienced in extracting a polypus from the uterine cavity after it has been detached. Prof. Simpson mentions one case in which the os uteri was divided to allow the possibility of extracting a polypus of the size of a filbert. Vulselli with short teeth do not always hold, and those with long teeth are difficult to open in the narrow uterine cavity; besides, there is danger that these latter may catch in the uterine walls.

(TO BE CONCLUDED.)

